# Factors associated with recent iodine intake level among household food handlers in Southwest Ethiopia: a cross-sectional study

**DOI:** 10.1186/s12905-023-02516-8

**Published:** 2023-07-04

**Authors:** Agize Asfaw, Mifta Behailu, Abdu Oumer, Tigist Gebremariam, Kenzudin Asefa

**Affiliations:** Department of Public Health, College of Medicine and Health Sciences, Wolkite University, Welkite, P.O. Box: 07, Ethiopia

**Keywords:** Ethiopia, Food handlers, Iodine deficiency, Iodine intake, Iodized salt

## Abstract

**Background:**

Iodine deficiency is a global public health threat, affecting an estimated two billion people. The median urinary iodine concentration is more reliable in determining recent iodine intakes and the risks of iodine deficiency. Therefore, this study was aimed to identify the factors associated with recent iodine intake level using median urinary iodine concentration as an indicator among household food handlers in southwest Ethiopia.

**Methods:**

A community-based survey was conducted with selected households using a pretested interviewer-administered questionnaire in southwest Ethiopia. A 20-gram sample of table salt and a 5 ml causal urine samples were also collected and analyzed using rapid test kit and a Sandell-Kolthoff reaction, respectively. A salt iodine concentration above 15 ppm was classified as adequately iodized and a median urinary iodine concentration between 100 and 200µgl^− 1^ was considered as adequate iodine intake. A bivariable and multivariable logistic regression model was fitted. Crude and adjusted odds ratios with their 95% confidence levels were reported. Associations with a p-value ≤ 0.05 were used to declare statistical significance.

**Results:**

A total of 478 women were included, with a mean age of 33.2 (± 8.4 years). Only 268 (56.1%) of the households had adequately iodized salt (> 15 ppm). The median urinary iodine concentration (interquartile range) was 87.5 µg l^− 1^ (45.6-107.6). In a fitted multivariable logistic regression model (p-value = 0.911), illiterate women (AOR = 4.61; 95% CI: 2.17, 9.81), poorly iodized salt in the household (AOR **=** 25.0; 95% CI: 13–48), salt purchased from open market (AOR = 1.93; 95% CI: 1.0, 3.73) and women who do not read the label during purchasing the salt (AOR = 3.07; 95% CI: 1.31, 7.17) were important predictors of the risk of Iodine deficiency.

**Conclusion:**

Despite public health efforts to improve iodine intake, its deficiency is still a major public health problem among southwest Ethiopian women.

## Background

Iodine is integral to the synthesis of thyroid hormones for the regulation of metabolism, growth, and development in human beings [1, 2]. The dietary sources of iodine are diverse, but the majority of the food sources are deficient in iodine, except sea foods and food sources from soil with adequate iodine content. Moreover, iodine nutrition is dependent on the iodine content of the soil, where plants and animals get nourished [3–5]. Poor intake of seafood rich in iodine, people living in highlands with frequent soil erosion, and habitual intakes of goitrogens which reduce iodine uptake predispose to prevailing iodine deficiency disorders (IDD) [6].

Globally, an estimated 2 billion people are victims of IDD. Iodine deficiency (ID) is strongly linked to poor physical growth and significant cognitive capacity reduction in iodine deficient individuals [7]. Furthermore, more than five million children are victims of the severe form of IDD, accompanied by severe mental and physical growth deficits. Maternal ID is associated with an increased risk of malformed births, cretinism, miscarriage, and still births [2].

Thus, to alleviate the inherent ID in the soil and widespread IDDs, mandatory salt iodization has become an important public health strategy globally. Evidence showed that universal salt iodization significantly reduced clinical IDDs by 80% (20.5 million cases averted) and had a net economic gain of $33 billion due to improved cognition by 2019 [8]. A recent report showed that 89% of the world population consumes iodized salt despite IDD being prevalent. In addition, modeling showed that 4.8 million children still suffer from IDDs [8]. Similarly, in Ethiopia, 55% of children had goiters and their recent iodine intake level was low (96 µg l^− 1^) [9].

Even though, the implementation of salt iodization programs over the past decades to prevent and control IDDs were promising [10–14], the problem remains a major threat to the health and development of populations [15]. The impact is still above the threshold among children and women in developing countries, particularly in Ethiopia [16–20]. A recent national survey indicated that 89% of households use iodized salt. However, only 26% of households get adequately iodized salt [21]. This is suggestive that there are multifaceted problems in manufacturing, transporting, handling, and utilizing iodized salt. In addition, a strong monitoring and evaluation of iodization program usually requires periodic evidence.

To monitor the overall progress of IDDs, the World Health Organization recommends MUIC to reliably assess the recent iodine intake and assess the move towards the targets [2]. This indicator is a population-level indicator for inadequate iodine intakes. As the goiter takes a longer time period to recover, the MUIC is the best indicator of recent iodine status for children, pregnant women, and lactating women [22, 23].

Previous studies [24–28] mainly target school-age children and assess the overall prevalence of goiter, which is not a sensitive indicator to assess the recent iodine status [4]. Having timely evidence on the level of salt iodization and the iodine intake of household food handlers better informs national and regional planners to track the progress toward the target and helps to sustainably address IDDs [29, 30]. Thus, the purpose of this study was to determine the factors associated with recent iodine intake among household food handlers in southwest Ethiopia.

## Materials and methods

### Study setting and design

A community-based cross-sectional study was conducted in southwest Ethiopia from June 15 to July 15, 2021. The study site is located in the Guraghe zone, 197 km away from Addis Ababa, the capital city of Ethiopia. It is located at an elevation of 1500 to 2300 m above sea level, with an average environmental temperature ranging from 13 to 25^0^ C. It has 42 rural administrative kebeles (smallest administrative units in a district) with a total of 28,992 households and a total population of 149,976.

### Population and eligibility criteria

The current study was conducted on a random sample of households in the southwest of Ethiopia. Adult women aged 18 years and older in the selected households who were primarily involved in the preparation of food and handling salt within the households were included.

### Sample size and sampling procedures

The minimum sample size for this study was estimated using sample size estimation for assessing the prevalence of a particular outcome, and the epiinfo module for sample size calculation was used to estimate the sample size for the objective. Taking the prevalence of adequate salt iodization (26%) [31], 95% confidence level, 5% maigin of error, 10% non-response rate, and a design effect of 1.5 to account for possible hetrogenity, a total of 478 households were needed for this study. A probability sampling technique with a proper allocation method was used. First, the total sample was proportionally allocated to a randomly selected 12 kebeles from a total of 42 rural kebeles using simple random sampling. The allocated samples were selected from each kebele using a systematic sampling method with sample intervals. The sampling interval (K) was determined by dividing the total number of households in the kebele by the allcated sample. After selecting the first house randomly, houses at every sample interval were recruited. An adult female member of the household who was mostly responsible for food preparation and salt handling was included in the study [32].

### Data collection methods

A combination of face-to-face household interviews, samples of table salt, and mid-stream causal urine samples were used in this study. The interviewer-assisted interview was conducted by four trained clinical nurses with the randomly selected woman using a pre-tested structured questionnaire. The questionnaire asked about respondents’ socio-demographic characteristics as well as their knowledge and practice of iodized salt handling and utilization [33, 34]. In addition, information on household assets was collected based on the demographic health survey tool to assess the household wealth index. Two trained laboratory technicians took 5 milliliters of casual urine from each respondent, using a labeled plastic bottle with a screw cap and handling it in an ice-packed cool box [35]. The collected urine was transported to the nearest primary hospital laboratory for storage in a refrigerator at -4^o^c until it was transported to the Ethiopian public health institute for analysis. The analysis was done using the modified Sandell-Kolthoff reaction, which can reliably quantify the MUIC. The iodine status of respondents was classified by using World Health Organization (WHO), United Nations Children’s Fund (UNICEF) and Iodine Global Network (IGN) recommended cut-off points for UIC. Accordingly, UIC value > 300 ?g l^− 1^ (excessive iodine intake), 200–299 ?g l^− 1^ (more than adequate intake), 100–199 ?g l^− 1^ (adequate intake), 50–99 ?g l^− 1^ (mild ID), 20–49 ?g l^− 1^ (moderate ID) and < 20 ?g l^− 1^ (severe ID) [4, 23].

Finally, during data collection, interviewers asked every respondent to provide about 20 g of salt used for cooking. The collected sample was labeled with a code and submitted to the principal investigator. Then, the sample was tested by a trained laboratory technician using a rapid test kit for iodine (MBI Kits International) for its iodine content. The iodine concentration was recorded as 0, < 15, or ≥ 15 PPM as per the standard procedure [4, 26, 36].

### Operational definitions

In this study, we defined adequately iodized salt when the rapid test kit chart color turned to deep blue during testing (i.e. the iodine content in the sample salt is at least 15 parts per million). Proper practice of handling iodized salt refers to adding salt to cooking food at the end or right after cooking in the last 24 h. While the MUIC is classified based on the WHO population level iodine intake classification, where a MUIC above 200 µg l^− 1^, 100–200 µg l^− 1^, and below 100 µg l^− 1^ is operationally defined as having above normal, normal, and inadequate iodine intakes, respectively based on our data [4].

### Data processing and analysis

The data was pre coded, cleaned, and entered into Epi Info version 3.5.4 and exported to SPSS version 23 software packages for statistical analysis. Then it was presented in statistical tables, graphs, and frequencies. In addition, mean, median, and standard deviation were used as needed. The household asset variables were coded as “Yes” and “No” and the wealth index was derived from individual household assets using principal component analysis (PCA), and the factor scores were ranked into three terciles: wealthiest, medium, and poorest. Items which fulfill the assumptions of PCA were considered for the analysis [37–40].

A binary logistic regression model was fitted to identify factors associated with the risk of ID among adult women. Variables with a p-value ≤ 0.2 in the bivariable analysis were fitted into the multivariable logistic regression analysis. The crude (COR) and adjusted odds ratios (AOR) were reported with a 95% confidence interval (CI). Associations with a p-value of ≤ 0.05 were declared statistically significant. Multicolinearity and effect modifications were checked accordingly [41, 42]. Multicolinearity was evaluated using the variance inflation factor (above 10) and inflated standard error (above 2), where no significant multicolinearity was noted [43].

## Results

### Socio-economic characteristics of respondents

In this study, a total of 478 households were included, with a mean age (standard deviation) of respondents of 33.2 years (± 8.42), where the majority, 396 (82.8%), were married. More than two-thirds (69.0%) of respondents were illiterate without any formal education. In addition, based on the wealth ranking, 50% of the participants were in the medium wealth category, while 24.3% of them were in the poorest wealth status (Table 1).

**Table 1:** Socio-economic characteristics of the study participants in southwest Ethiopia (n = 478)

Variables	Category	Frequency	Percent
Age in years	18–34 35 and above	250 228	52.3 47.7
Marital status	Single Married Widowed Divorced	82 375 13 8	17.2 78.5 2.7 1.6
Educational status	No formal education Can read and write ^¶^Formal education	330 76 72	69.0 15.9 15.1
Occupation	House wife Merchant Student Farmer	322 73 61 22	67.4 15.3 12.8 4.6
Family size	< 5 >=5	240 238	50.2 49.8
Wealth index	Poor Medium Rich	116 239 123	24.3 50.0 25.7

^¶^Formal education = includes primary, secondary and college level educations

### Household salt iodization level

Based on the iodine level of the sampled salt, 43.9% (95% CI: 41.5–46.3%) of the households had inadequately iodized salt (iodine content < 15ppm) for human consumption. Among these, 170 (63.0%) were unpacked salt users. On the other hand, the majority of the inadequately iodized salt users (83.0%) were using salt from unpackaged sources for household utilization.

### Recent iodine intake level

**Fig. 1:**
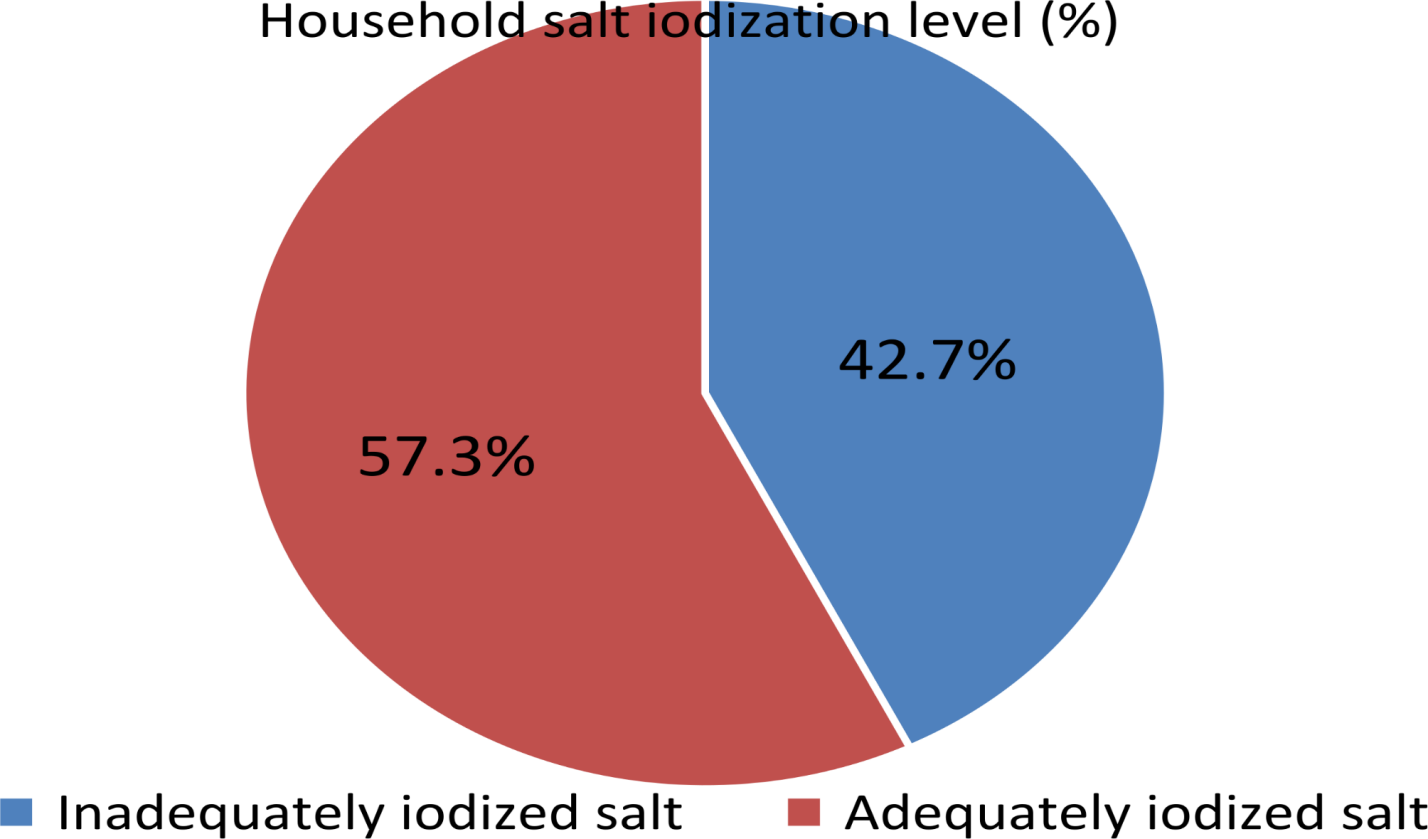
Iodine content of salt at household level in southwest Ethiopia, 2021

**Fig. 2:**
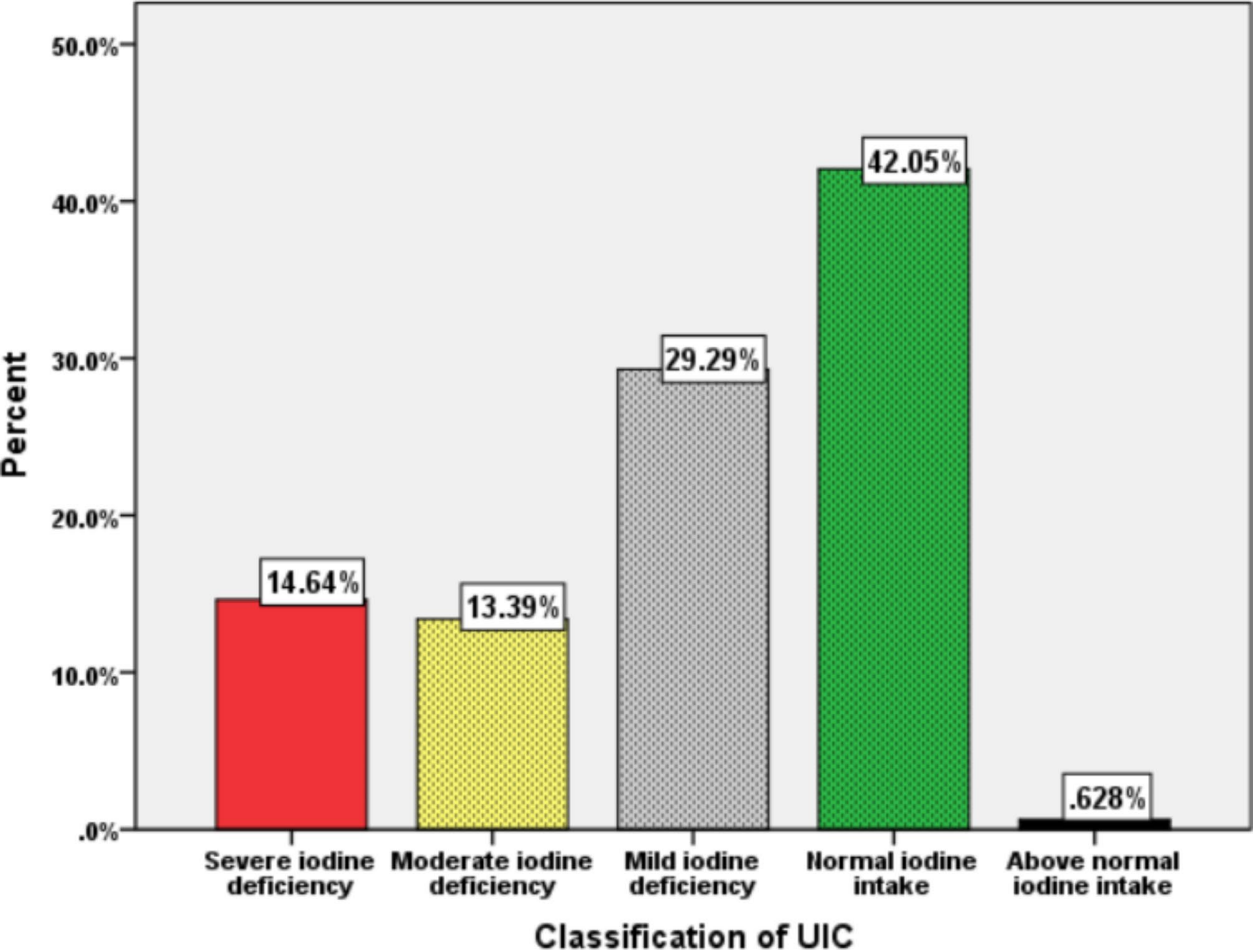
Recent iodine intakes of women at household level in southwest Ethiopia, 2021

The population level median UIC (IQR) for this study was 87.5 µg l^− 1^(45.6-107.6). Furthermore, 42.7% of women (95% CI: 40.4 to 45.0%) had inadequate iodine intakes (Fig. 1), leading to imminent iodine deficiency. A statistically significant moderate correlation was observed between household food handlers’ knowledge of proper handling of iodized salt and recent iodine intake (r = 0.19, p < 0.001). In addition, there was a statistically significant weak negative correlation between the recent iodine intake indicator and unpackaged salt type (r = -0.26, p < 0.001) and food handlers’ practice in handling iodized salt (r = -0.23, p < 0.001). The detailed description of recent iodine intakes of women as classified by UIC level was presented in Fig. 2.

### Factors associated with recent iodine intakes

We ran a binary logistic regression analysis to assess the crude association between each factor and the risk of ID (low MUIC) among women. In the bivariable analysis, age, educational status, wealth index, salt type, iodine status of household salt, knowledge of iodine use, proper handling practices of iodized salt, place of purchasing iodized salt and methods used to check iodine content of salt during purchasing were significantly associated with the ID. Those older women, aged above 35 years (COR = 1.98; 95% CI: 1.37–2.85), illiterate (COR = 3.75; 95% CI: 2.41–5.84) were two and four times more likely to have inadequate iodine intakes as compared to their counterparts. Furthermore, women from low socioeconomic status (COR = 2.64; 95% CI: 1.56–4.48) and using unpacked salt (COR = 3.03; 95% CI: 2.06–4.46) were positively associated with a 2.64 and 3.03 times increased risk of ID, respectively. Women with inadequate knowledge regarding iodine nutrition (COR = 2.27; 95% CI: 1.54–3.45), poor handling practices (AOR = 2.73; 95% CI: 1.84–4.04) and having poorly iodized salt in the house (COR = 16.3; 95% CI: 10.34–25.6) were more likely to have ID than the others (Table 2).

**Table 2:** Bivariate and multivariable logistic regression model showing the predictors of iodine deficiency (inadequate iodine intake) among women in southwest Ethiopia

Variable	Category	COR (95% CI) ^a, b^	AOR (95% CI)	p-value
Age	18–34 years	1	1	0.371
	>=35 years	1.98 (1.37, 2.85)	1.27 (0.75, 2.13)	
Educational status	Literate Illiterate	1 3.75 (2.41,5.84)	1 4.61 (2.17, 9.81)	0.001**
Occupation	Employed House wife	1 1.35 (0.91,1.99)	1 1.68 (0.94, 3.0)	0.078
Wealth index	Rich Medium Poor	1 1.75 (1.13, 2.71) 2.64 (1.56, 4.48)	1 1.62 (0.90, 2.91) 1.22 (0.58, 2.56)	0.602 0.109
Salt type	Packed Unpacked	1 3.03 (2.06,4.46)	1 1.02 (0.54, 1.91)	0.962
Salt iodine level	Adequately iodized ^c^	1	1	0.0001**
	Inadequately iodized ^d^	16.3(10.34, 25.6)	25.0(13, 48)	
Knowledgeable about iodine	Yes	1	1	0.441
	No	2.27 (1.54, 3.45)	1.28 (0.69, 2.37)	
Handle salt properly	Yes	1	1	0.532
	No	2.73 (1.84,4.04)	1.21 (0.66, 2.21)	
Source of salt	Retail shop Open market	1 3.65 (2.48,5.36)	1 1.93 (1.0, 3.73)	0.049*
Method to check iodine content	Read the label	1	1	0.010*
	Do nothing	2.16 (1.33, 3.53)	3.07 (1.31, 7.17)	

^a, b^*the outcome variable, recent iodine intake was operationalized in to*^*a*^*-MUIC below 100 µg l*^*− 1*^*and*^*b*^*-MUIC > = 100 µg l*^*− 1*^, *the iodine content of the household salt sample was defined as*^*c*^*-when the salt iodine concentration is at least 15 ppm (adequately iodized salt) and*^*d*^inadequately iodized salt, when the iodine content of the salt is below 15 ppm as indicated in color change of the test kit. * Refers to association with a p-value below 0.05 and** refers to p-value below 0.01

A possible interaction, multicollinearity, and confounding effects were explored through appropriate statistical procedures, and no statistically significant effect of modification and collinearity was observed. While the confounding effects were explored further and presented in Table 2 above. The multivariable logistic regression model was fitted adequately (p-value for Hosmer-Lemeshow test = 0.911) where educational status, salt iodine level, place where salt was puchased and methods used to check iodine content of salt during purchasing were important predictors of inadequate recent iodine intakes. Those respondents without formal education (AOR = 4.61; 95% CI: 2.17–9.81) and do not read the label on the pack during purchasing the salt (AOR = 3.07; 95% CI: 1.31–7.17) were 4.6 and 3.1 times more likely to be affected by ID respectively. Moreover, women who bought the salt from open market (AOR = 1.93; 95% CI: 1.0–3.73) were about 2 times more likely to be affected by ID than those who bought from retail shop. Above all, the presence of inadequately iodized salt in the household significantly increased the risk of ID among women (AOR = 25.0; 95% CI: 13.0–48.0) in this study (Table 2). Women who possess inadequately iodized salt in their house were 25 times more at risk of ID compared to their counterparts.

## Discussion

The findings of this study showed that 56.1% of households used adequately iodized salt while 42.7% of women had inadequate recent iodine intake (MUIC < 100 µg l^− 1^), with a prevailing risk of ID among women in southwest Ethiopia. It is recommended that MUIC taken from a representative sample is a good indicator of the current iodine status in the general population and predict the risk of imminent ID in more accurate way than goiter prevalence [4].

In this study, the MUIC was found to be 87.5 (95% CI: 45.6-107.6) µg l^− 1^, which is far below the adequate level. The finding of this study is higher as compared to studies conducted in other parts of southern Ethiopia (MUIC = 1.9 µg l^− 1^) [17], Northwest Ethiopia (MUIC = 39.9 µg l^− 1^) [20] and west Gojam in Ethiopia (MUIC = 5 µg l^− 1^) [26]. It is also better as compared to studies conducted in other countries like Viet Nam (MUIC = 70 µg l^− 1^) [18] and northern Morocco before introduction of iodized salt (MUIC = 17 µg l^− 1^) [12]. This difference might be attributed to the practice of salt iodization and socioeconomic status of the community where the study was conducted. Even though the recent iodine intake is marginal, it is still above the reports from the mentioned different studies. However; complemented by higher consumption of goitrogenic factors (kale, cabbage, lettuce and beets) and prevailing poor handling practices of households, the risk of ID is high among women in the study area. In addition, the prevailing vitamin A deficiency [6], selenium deficiency [44], and zinc deficiency [45] will interact and significantly increase the risk of ID. This could potentially increase the risks of adverse pregnancy outcomes, severe cognitive losses and greater burden of ID among women [46].

Several factors were found to be associated with ID. Women without formal education, older age, from low socioeconomic status, and those who purchase the salt from open market for household consumption were associated with lower level of recent iodine intake and higher risk of ID. The importance of education in the prevention of ID is further elaborated by the fact that respondents who read the labeled information on the packed salt during purchasing were less likely exposed to ID. In addition, women from low socioeconomic status tend to have a poor access to education and employment, where they have a limited information and knowledge on iodine nutrition, proper handling, preparation and use of iodized salt. The finding of this study is in line with previous studies conduced in Ethiopia and elsewhere [26, 27, 33, 34, 47, 48]. One study also reported that maternal schooling is significantly associated with the risk of micronutrient deficiency where it can be considered as nutrition-sensitive intervention to adress ID [49]. Samson et al. [50] also showd that educated women tends to have a higher iodine intake and MUIC (p < 0.0001) as compared to uneducated one. It is also supported by the finding that knowledge of women about iodized salt had shown statistically significant positive correlation with recent iodine intake levels. This will in turn increase the differential risk of ID among such segment of the population and helps in targeting interventions.

It is known that iodized salt is a dominant source of the daily iodine requirement for human. The household availability of adequately iodized salt (56.1%) was comparable with studies conducted in Southeast (56.6%) and southern Ethiopia (58.2%) [10, 28]. While the result was lower than studies conducted in other parts of Ethiopia like Assella (62.9%) [51], Mecha district (63.3%) [14], and Northeast Ethiopia (68.8%) [18]. It is also lower when compared to studies conducted in other countries such as Sri Lanka (91.2%) [25], South Africa 75% [23], Egypt (78%) [3]. Our study also indicated that the presence of adequately iodized salt (AOR = 25.0; 95% CI: 13–48) significantly associated with a higher iodine intake and reduced risk of ID. This low availability of adequately iodized salt coupled with improper handling practice further aggravate the iodine intake and risk of ID. It is evidenced by the strong correlation between household salt iodization with MUIC (r = 0.60, p < 0.001). It is plausible that households having adequately iodized salt in their house tend to have higher UIC level than those who do not have adequately iodized salt [5, 34]. However, the pack status of the salt iodine did not determine the actual iodine intakes as intake is complex interaction between availability, level of iodine loss due to poor handling and other factors.

In addition, women who consume salt from open market (AOR = 1.93; 95% CI: 1.0-3.73) had an increased risk of ID than from retail shops. This is strongly associated with the fact that iodine is sensitive at exposure to sunlight and open air, which tends to be volatile and the amount available for human consumption would be low. Previous studies also showed that buying salt from open market tends to increase the iodine loss and it significantly affects the iodine level in the salt for human consumption [14, 24, 36, 52]. This emphasizes the need to handle iodized salt from production, transportation, and storage and food preparation to retain maximum possible iodine content for human consumption. It could be addressed through awareness creation schemes targeting transporters and sellers in open market to handle the salt properly.

The findings of this study have wide practical implications. Although the government of Ethiopia is implementing salt iodization program over a decade, still ID is a major public health problem among women and low iodine intake is more prevalent [53]. There are still differences in the way individuals understand its importance, how they practice, and actually utilize it which affects the intake level. In addition, ID is associated with poor economic status of women, limited employment opportunity and low educability that limit information access on iodine nutrition. This gap indicates the need for additional and targeted actions such as behavioral change communication and education on proper handling and utilization of iodized salt and giving priority for most needy groups like women and children.

Finally, this study is not free of limitations. One of the limitations is the household salt iodization level was based on qualitative approach which may not actually reflect the level of salt iodization. If Iodometric titration test was done, it would have estimated the iodine content of the salt samples quantitatively. In addition, the possibility of seasonal variations in the iodine intake from dietary sources in addition to iodized salt. Furthermore, respondents might have a potential social desirability bias in responding to questions during interview. They may tend to give iodized salt although they use non iodized salt in the majority of the time.

## Conclusion

Iodine deficiency is a public health problem of household food handlers in southern Ethiopia and the availability of adequately iodized salt at household is far below the recommendation. This means, the universal salt iodization program, which was aimed to ensure access to iodized salt by all people is not appropriately implemented. Therefore, it is crucial to intensify efforts in the implementation of universal salt iodization program focusing on most vulnerable groups. In addition, targeted behavioral change communication and information education communication is needed regarding proper handling practice of iodized salt in this area.

## Data Availability

All relevant data are within the manuscript and if additional data is requested, the corresponding author is ready to submit or discuss on ambiguity.

## Abbreviations

A/COR: Adjusted/Crude Odd Ratio
CI: Confidence Interval
IGN: Iodine Global Network
IDD: Iodine Deficiency Disorders
ID: Iodine Deficiency
MUIC: Median Urinary Iodine Concentration
PCA: Principal Component Analysis
WHO: World Health Organization
